# Evaluating the impact of social and behavior change communication intervention on improving documentation practices among healthcare workers in southern Nigeria: a before and after study

**DOI:** 10.3389/fpubh.2025.1462944

**Published:** 2025-04-09

**Authors:** Chinwe Eze, Ngozi Murphy-Okpala, Okechukwu Ezeakile, Joseph Chukwu, Ngozi Ekeke, Chibuike Agu, Ifeyinwa Ezenwosu, Sode Matiku, Beatrice Kirubi, Anthony Meka, Martin Njoku, Francis S. Iyama, Edmund Ossai, Akpan Bassey, Johnson Babalola, Obioma Chijioke-Akaniro, Charles Nwafor

**Affiliations:** ^1^Department of Programs, RedAid Nigeria, Enugu, Nigeria; ^2^Department of Medical, DAHW German Leprosy and Tuberculosis Relief Association, Würzburg, Germany; ^3^Department of Community Medicine, Alex-Ekwueme Federal University Ndufu-Alike, Abakaliki, Nigeria; ^4^Department of Community Medicine, University of Nigeria Teaching Hospital Enugu, Enugu, Nigeria; ^5^New Dimension Consulting, Dar es Salaam, Tanzania; ^6^STOP TB Partnership, Geneva, Switzerland; ^7^Department of Community Medicine, Ebonyi State University, Abakaliki, Nigeria; ^8^Akwa Ibom State TB, Leprosy, and Buruli Ulcer Control Program, Uyo, Nigeria; ^9^Oyo State TB, Leprosy, and Buruli Ulcer Control Program, Ibadan, Nigeria; ^10^National TB, Leprosy, and Buruli Ulcer Control Program, Abuja, Nigeria

**Keywords:** social and behavior change communication, effectiveness, documentation practices, healthcare workers, Nigeria

## Abstract

**Background:**

Proper documentation is very essential to healthcare practice. It is vital for continuity of care and communication among healthcare workers. This study was designed to determine the effectiveness of the application of Social and Behavioral Change Communication strategy to improving documentation practices among healthcare workers in two states in Nigeria.

**Methods:**

A longitudinal study with a pre-post-intervention design was utilized. An audit of documented specimen examination request forms was conducted at two-month intervals. The initial audit served as baseline. Subsequent audits were to monitor progress and assess impact of intervention. For each audit, a minimum of 10 facilities was purposively selected across the two states, including an average of five GeneXpert sites and six health facilities. Based on availability, a maximum of 50 specimen examination request forms were consecutively selected per GeneXpert site or facility for audit. The interventions included modification of the specimen examination request form, training and educational sessions, regular audit of forms, progress monitoring, and feedback.

**Results:**

There was an increase in the proportion of each variable meeting the documentation standard across the four audit rounds. This progress was observed across the four audit rounds for most of the variables, with the most substantial improvement recorded in the fourth audit. In all, there was a significant improvement in the proportion of each specimen examination form that met the documentation standards (*p* < 0.001). These variables included detailed address of patient, patient phone number and phone number of person requesting the examination. Variables on the revised form which showed significant changes across the four audit periods included name and phone number of next of kin (*p* < 0.001).

**Conclusion:**

The SBCC intervention markedly enhanced accurate and comprehensive documentation of specimen examination request forms among frontline healthcare workers. Key components, such as repeated training sessions, education emphasizing the benefits of sound documentation practices, consistent supervision, monitoring, feedback, and ensuring a sufficient supply of forms, collectively played integral roles in maximizing the effectiveness of the intervention.

## Introduction

Good documentation is a critical component of healthcare practice and its importance cannot be overemphasized. It is vital for patient safety and quality care as well as protecting the healthcare worker against or in case of medical litigation (1). Proper documentation is necessary for continuity of care, communication among healthcare workers and patients concerning diagnosis, laboratory results, treatments and outcomes, education, research, and funding purposes (2–5).

Documentation in healthcare settings can take the form of electronic or paper-based records. While the electronic medical record system (EMR) has almost replaced the paper-based system in developed countries, the norm in many low-and-middle income countries remains the paper-based system. Irrespective of the format, documentation must be patient-centered, complete, accurate, relevant, clear, timely and confidential (2).

While acknowledging the critical importance of maintaining proper documentation, gaps still exist globally in healthcare documentation practices. For instance, health professionals documentation practice is reported to be <50% in Iran (6), 47% in England (7), 47.8% in Ethiopia (8), and as low as 33.3% in Indonesia (9). Other studies spanning developed and developing countries consistently underscore limited documentation practices in patient care (7, 10–13). In Nigeria, several literature and practice-based observations have also shown widespread deficiency in the knowledge and practice of proper documentation among healthcare workers (14–17). One study quantifying documentation practice showed that only 44% of health professionals had good knowledge and documentation practice (2).

Poor documentation practices highlighted in these studies encompass issues such as incomplete or missing records, illegible writings, and inaccurate and poor-quality records. These result in poor patient management arising from misdiagnosis, care omissions, incorrect care delivery and lack of evidence-based decision-making along the patient care pathway (18). Ultimately, these issues contribute to adverse patient outcomes, medical errors, and in severe cases, patient deaths (19).

Studies have shown several factors as causes of poor documentation. These include shortage of staff, high workload, poor motivation and attitude of health care workers, limited knowledge about documentation requirements, lack of awareness, insufficient time, infrequent supervision, monitoring and evaluation, lack of training, and shortage of recording materials (11, 15, 16, 20, 21). To address these challenges, recommendations included continuous training and education, regular audits of medical records, and ensuring sufficient supply of recording materials (14, 16). Additionally, fostering a positive attitude among healthcare professionals toward documentation is crucial. This can be achieved by emphasizing the value of documentation in their professional practice, emphasizing the implications of poor documentation, and providing motivation for engaging in effective documentation activities (18, 22).

Social and Behavioral Change Communication (SBCC) entails an intentional approach to positively influence norms, attitudes, beliefs, and behaviors and is widely used in different fields. It is therefore, a potentially effective strategy to improve attitudes and documentation practices among health professionals. This is underscored by its demonstrated effectiveness in several behavior change interventions such as quitting smoking, dietary adjustments and promotion of exercise (23), use of insecticide-treated nets (24–26) and improvement of infant and young child feeding practices (27). SBCC adopts a strategic process in the design and implementation of communication interventions aimed at fostering positive changes in attitudes, beliefs, and behaviors within a target group.

This paper reports the effectiveness of the SBCC strategy applied to improve documentation during the TB REACH Wave 9 project implemented by RedAid Nigeria and sponsored by the Stop TB partnership.

## Methodology

### Intervention area and setting

The intervention was conducted between December 2022 and June 2023 as part of the 18-month TB REACH Wave 9 project titled “Catalyzing improvements in drug-resistant tuberculosis (DR-TB) care in Nigeria: A Sustainable Patient-centered approach” which was aimed at improving linkage to care by reducing pre-treatment loss to follow up (PTLTFU) for DR-TB patients. The project was implemented under routine programmatic conditions in two states in southern Nigeria (Akwa Ibom and Oyo). There are a total of 1,209 Directly Observed Treatment Short-course (DOTS) facilities (Akwa-Ibom:538, Oyo:671) and 28 GeneXpert machines (Akwa-Ibom:13, Oyo:15) in the project states. Each GeneXpert site serves an average of 40 DOTS facilities based on proximity and functionality.

Nigeria is one of the leading contributors to the global gap between estimated TB incidence and notified new TB cases (28). Additionally, a significant gap exists between the estimated incidence of DR-TB and the actual reported cases. According to the WHO Global Report, only 14% (2,975) of the estimated 21,000 DR-TB cases were officially notified in 2021 (28). Despite these alarming figures, Nigeria faces another substantial challenge with DR-TB diagnosis and enrollment, which is a significant pre-treatment loss to follow-up (PTLTFU) as high as 26% in 2021. Routine surveillance data ranked Oyo and Akwa-Ibom states among the top 10 contributors to PTLTFU in Nigeria in 2021.

One of the key factors contributing to high PTLTFU in the two states as well as in Nigeria as a whole, is the difficulty in tracing patients after the diagnostic test (GeneXpert MTB/Xpert/Rif) results are out. This pertains to patients without valid contact details, thereby impeding communication of test results and the DR-TB treatment enrolment process.

In a bid to close the TB treatment coverage gap, active case finding is conducted in facilities by healthcare workers and ad-hoc staff (TB screening officers- SOs), and in the community, by community healthcare workers. On identification of a presumptive TB case, an appropriate specimen is collected on the spot and a specimen examination request form is filled capturing biodata variables such as patient name, age, sex, phone number and address. Other details required include the type of examination requested, type of specimen collected, name and contact details of the healthcare worker requesting the examination, health facility of the requesting staff and state where the health facility is located. These details are also captured in the National TB presumptive case register within the DOTS facility where the patient is identified. If the patient is identified in the community, the person is registered in the nearest DOTS facility. The sample alongside the specimen examination request form is taken to the nearest functional GeneXpert site for processing. Often, the results of the diagnostic tests are written on the specimen examination request forms and sent back to the requesting health facility for necessary action based on the outcome of the tests and subsequent documentation.

Upon detection of rifampicin resistance in the laboratory results, an automated messaging system, GxAlert, is utilized to dispatch short message service (SMS) notifications. These notifications are intended for both the state program manager and the state Drug-Resistant Tuberculosis (DR-TB) focal person, as outlined in the National DR-TB guidelines. The purpose of this automated communication is to expedite the responsiveness of the health system, ensuring the prompt linkage of diagnosed patients to the necessary healthcare services post-diagnosis. Following the SMS notifications, the State DR-TB focal person takes the initiative to inform the Local Government Tuberculosis Supervisor (TBLS) who in turn contacts and invites the patient for baseline investigations in preparation for enrollment into the treatment program. However, due to poor documentation of relevant details including patient contact details in the specimen examination request form, it is difficult to track some of these patients to provide further services. While the project engaged the services of the Global Fund (GF) funded community-based organization (CBO) for patient tracking within the community as originally planned in the project, their efforts were largely frustrated by insufficient contact details.

To reduce the number of patients who could not be tracked due to poorly documented contact details and improve linkage to treatment, we developed and implemented an additional intervention – SBCC, a few months into the project.

### Theoretical bases to inform the SBCC intervention

The SBCC content and intervention process were guided by concepts and principles drawn from the Information-Motivation-Behavioral Skills (IMB) model of behavioral change. While the model’s origin lies in responding to HIV epidemics (29), its constructs are considered generalizable determinants of health behavior (30). In this study, the model provided a structured framework for understanding and improving healthcare workers’ documentation practices by addressing their knowledge, motivation, and ability to implement proper documentation procedures. Additionally, it highlighted system-level support through supervision and monitoring.

Key constructs of the IMB model:

- Information: regarded as the primary prerequisite for behavior change (29, 31). Communicating what to document and how to document is crucial to ensuring a clear understanding of expected behavior to inform decision-making.
- Motivation: encompasses both personal and social motivations. Whereas personal motivation includes beliefs about the intervention outcome and attitudes toward a particular health behavior, social motivation deals with the individual’s perceived social support for engaging in the required behavior (32, 33). In this case, this includes the perceived benefits of good documentation practices for both the patient and the health worker’s professional practice, as well as the perceived adverse effects of poor documentation. Social motivation involves the health worker’s perception of social support from the State, the LGA TB program team, and the project implementing organization. This includes the availability of these individuals to regularly remind, supervise, and encourage health workers, along with providing relevant documentation tools.
- Behavioral skills: to promote behavioral change, the IMB model emphasizes the enhancement of an individual’s ability/skills to enact the behavior and increase perceived self-efficacy for it (33). To improve quality documentation skills and inspire confidence, the intervention components included training, regular monitoring, feedback, and mentoring.

Essentially, the constructs are interrelated as the presence of one could be a precursor to the other. For instance, an individual adequately informed and motivated individual will work toward developing and enacting the skills necessary to perform the required behavior (32).

### Brief conceptual framework and theory of change

**Figure 1:**
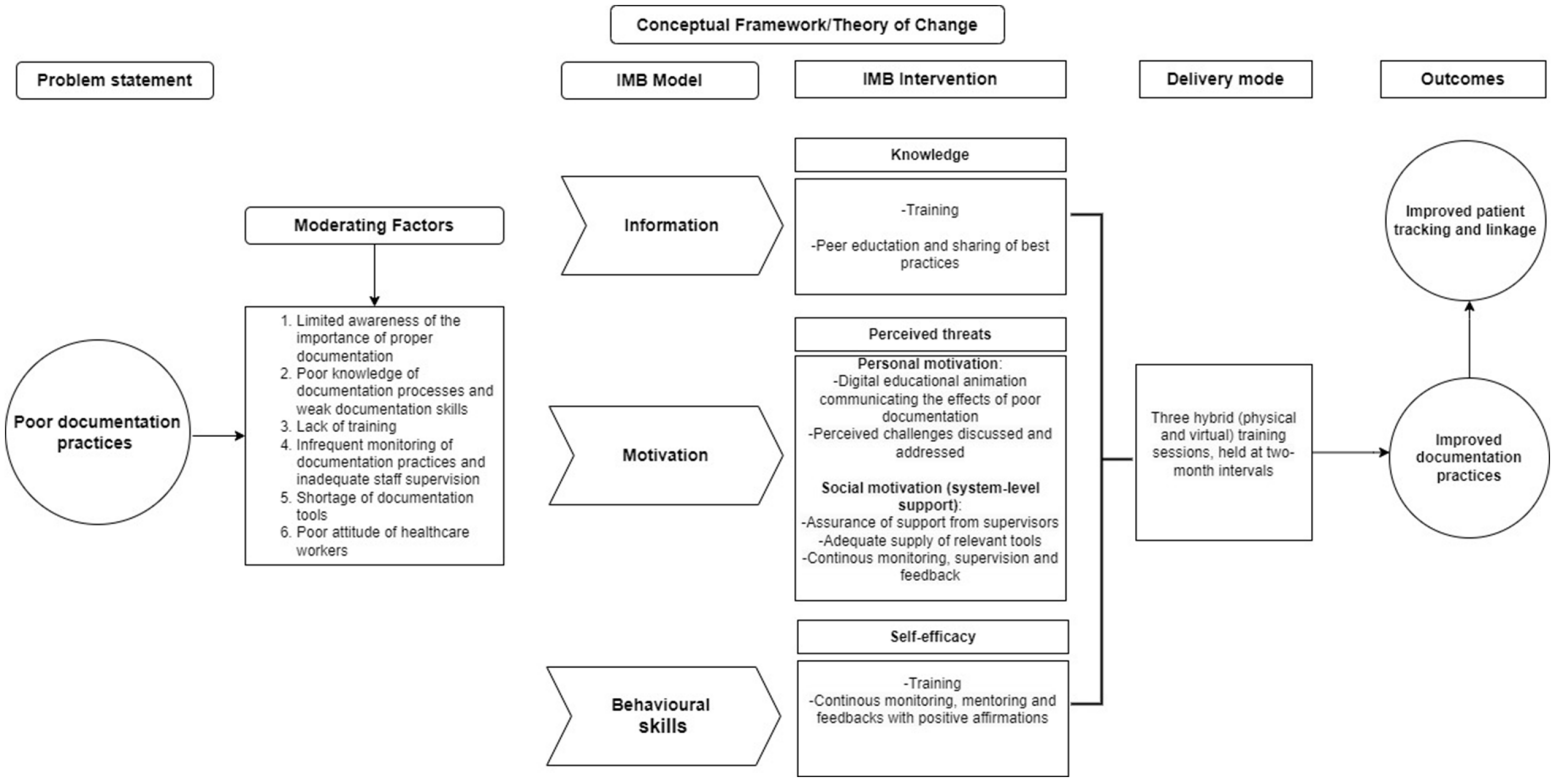
Conceptual framework/theory of change.

Figure 1 presents the conceptual framework plus the theory of change for the study. The conceptual framework serves as the foundation for understanding and addressing the core issue of poor documentation practices in healthcare settings. It identifies and outlines the moderating factors that contribute to this problem, such as limited awareness, insufficient training, inadequate supervision, and a lack of motivation among healthcare workers. By integrating the Information-Motivation-Behavioral Skills (IMB) model with key Social and Behavior Change Communication (SBCC) strategies—including targeted training programs, regular supervision, and structured feedback mechanisms—the framework provides a comprehensive approach to addressing these challenges.

The theory of change embedded within this framework posits that by enhancing healthcare workers’ knowledge, motivation, and documentation skills through SBCC interventions, significant improvements in documentation practices can be achieved. These improvements will be evidenced by the complete and accurate filling of the specimen examination form, a critical component of patient records. Over time, these changes are expected to lead to sustained improvements in patient tracking and linkage to care, ultimately contributing to better health outcomes and more efficient healthcare delivery.

### Intervention description

The interventions encompassed modification of the specimen examination request form, training and educational sessions, regular audit of forms, progress monitoring, and feedback that were implemented from November 2022 to June 2023.

- The existing specimen examination request form was modified to include extra variables such as next of kin details and the local government area (LGA) where the requesting facility is domiciled. The inclusion of next of kin details provides an alternative person to be reached in case the patient cannot be reached directly. The LGA information makes it easier to narrow down the search for a patient to a district and possibly a community, as well as the location of the requesting health facility, which is not possible where only state information is provided. The modified form was printed and distributed to the project states.
- Training and educational sessions with frontline health workers, including DOTS staff, TB screening officers and Community TB health workers: Training can be cost-intensive where hundreds of persons are involved. To make efficient use of the limited resources, a desk review of routine program data from the two states was conducted to identify the facilities that contribute 70–80% of the total presumptive TB cases in the states. Based on the Pareto principle, the underlying assumption is that improving the documentation practices of frontline health workers in these identified high-volume facilities would significantly impact the overall output of the state. A total of 217 DOTS facilities were thus involved in this intervention.

During the specified period, three interactive training sessions, spaced at 2-month intervals, each lasting 2–3 h, were conducted through a combination of in-person and virtual formats. The initial session was conducted in person, and organized in clusters across various states, while subsequent re-trainings took place virtually. On the average, 119 persons participated in a virtual session.

The sessions were comprehensive and adapted for a town hall meeting setting to encourage active participation. Participants were guided through the revised specimen examination request form. The significance and relevance of each variable on the form were explained, along with detailed instructions on how to accurately document them. For audiovisual aid, a digital educational animation was created to effectively convey the repercussions of inadequate documentation on patients, communities, and the broader health system. In addition, challenges to good documentation practices were elicited from the participants as well as their suggested solutions. Suggestions with broad consensus were collectively adopted. The meeting sessions also served as a space for peer education as participants were allowed to share best practices and how they handled challenges in their respective facilities.

iii. Progress monitoring and feedback: Changes in documentation practices were objectively monitored regularly. Feedback on progress and gaps was provided during the frontline health workers’ training sessions. Facilities demonstrating commendable performance were openly acknowledged to encourage and motivate others to strive for improvement.

### Study population

General healthcare workers in Akwa-Ibom and Oyo States.

### Study design

A longitudinal study with a pre-post-intervention design was utilized. The choice of a longitudinal approach allows for repeated measurements over time, enabling an evaluation of trends and changes in documentation quality. An audit of documented specimen examination request forms was conducted at two-month intervals. The initial audit, conducted in December 2022, served to establish a baseline. Subsequent audits were then carried out at two-month intervals, specifically in February, April, and June 2023, to monitor progress and assess the impact of the intervention.

For each audit round, a minimum of 10 facilities was purposively selected across the two states, including an average of five GeneXpert sites and six health facilities. Based on availability, a maximum of 50 specimen examination request forms were consecutively selected per GeneXpert site/facility for audit. In cases where the available forms were fewer than 50, all accessible forms were audited. As much as possible, the same facilities were used for the four audit rounds.

Each GeneXpert site holds specimen examination request forms from various facilities within its catchment area until diagnostic results are given out, making it possible to cover forms from more facilities within the catchment area during each audit round. For each round, forms from an average of 48 facilities were audited.

### Data collection tool and method

An Excel-based structured checklist formatted as tidy data was used for data collection. For each specimen examination request form, we gathered data on the State, facility name, and LGA. Additionally, a Yes or No response was chosen from a drop-down menu based on compliance with documentation standards for each key outcome variable. The outcome variables included the type of form (revised or old form), detailed address, patient phone number, name of next of kin, phone number of next of kin, LGA of the requesting facility, the name and surname of requesting staff and phone number of the requesting staff.

### Data analysis

Data was analyzed using Statistical Product and Service Solutions (SPSS), (IBM Company, New York, USA) version 25 software for both descriptive and inferential statistics and presented in a statistical table. Outcome measurement was done using descriptive analysis to compute the counts and proportion of each outcome variable that met the documentation standard for the different audit rounds. A simple line graph was used to visually assess the trend over time. Chi-squared test was used to assess statistical significance of compliance rates over four audit rounds, ensuring that observed improvements in outcome variables were not due to chance. Chi-squared statistical test was used because the outcome for each assessed variable is binary, different health records were assessed at each time point (ensuring independence across time points), and the goal was to compare proportions across the four time points. A *p*-value of <0.05 was considered statistically significant.

## Results

**Figure 2:**
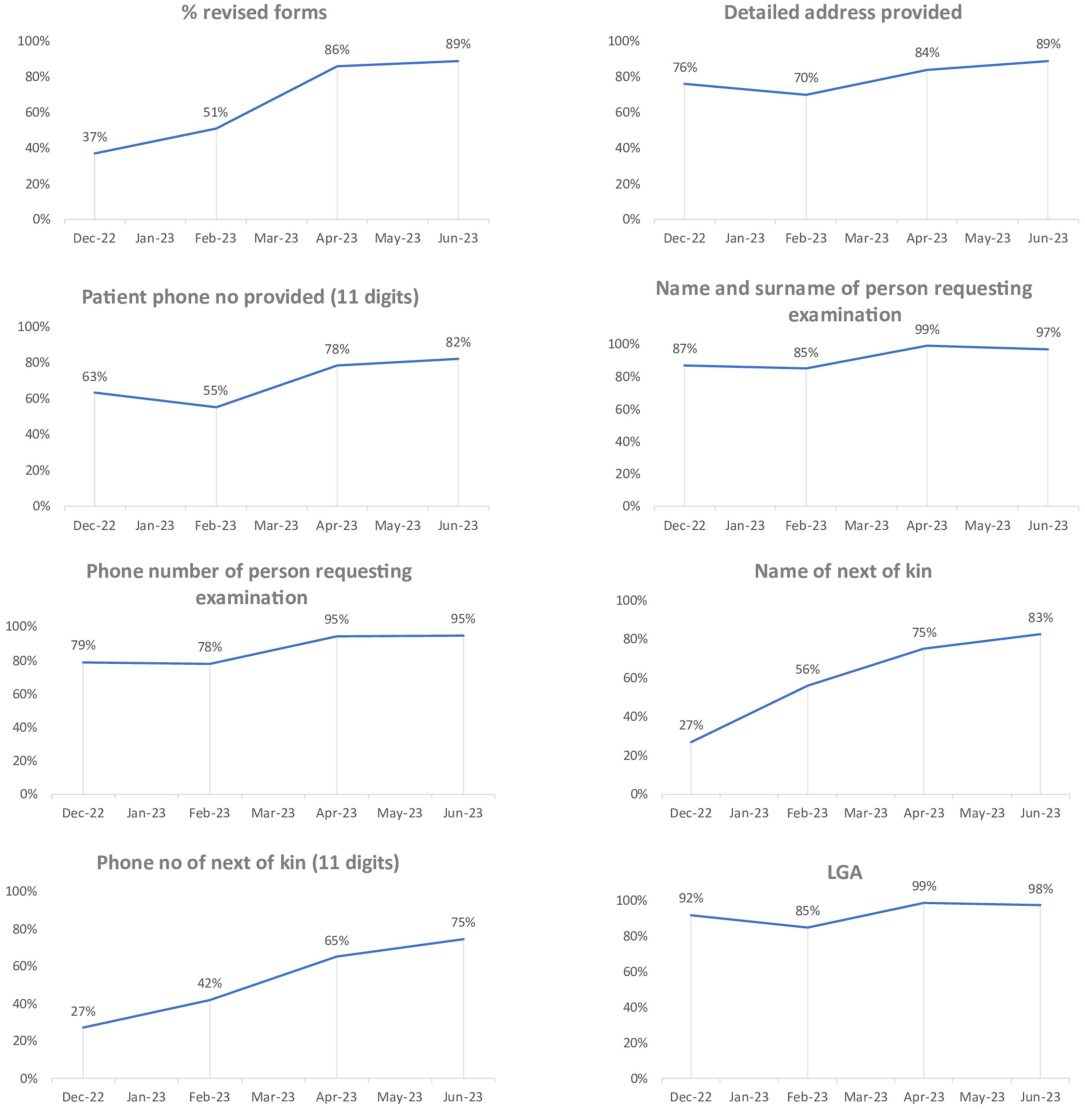
Graph showing the trend of the percentage of variables compliant with documentation standards.

Figure 2 provides a visual representation depicting the trend in the proportion of each variable meeting the documentation standard across the four audit rounds, whereas, Table 1 shows the statistical significance of observed changes in proportions for each variable.

**Table 1:** Statistical significance of observed changes in proportions for each variable.

Number and percentage of variables compliant with documentation standards					
Variable	December 2022 audit (Baseline)	February 2023 audit	April 2023 audit	June 2023 audit	*p*-value
Total number of forms reviewed	430	448	408	463	
Every entry has					
Type of form (Revised forms)	161 (37%)	229 (51%)	350 (86%)	412 (89%)	<0.001
Detailed address	327 (76%)	314 (70%)	344 (84%)	413 (89%)	<0.001
Patient phone number	270 (63%)	248 (55%)	320 (78%)	381 (82%)	<0.001
Name and surname of the person requesting the examination	376 (87%)	379 (85%)	402 (99%)	450 (97%)	<0.001
Phone number of the person requesting the examination	339 (79%)	351 (78%)	387 (95%)	438 (95%)	<0.001
Variables found only on the revised form					
Total number of revised forms	161	229	350	412	
Name of next of kin	44 (27%)	128 (56%)	263 (75%)	340 (83%)	<0.001
Phone number of next of kin	43 (27%)	97 (42%)	228 (65%)	307 (75%)	<0.001
LGA	148 (92%)	195 (85%)	345 (99%)	402 (98%)	<0.001

Overall, there is a significant improvement in the number and proportion of specimen examination forms that meet the documentation standards (*p* < 0.001). The progress observed has been consistent across the four audit rounds for most of the variables, with the most substantial improvement recorded in the fourth round.

Despite a marginal 1% reduction in the proportion of forms accurately documenting the Local Government Area (LGA) and a 2% decrease in the proportion of forms with properly recorded name and surname of the person requesting the examination in the fourth round compared to the third-round figures, these rates still surpassed those observed in the initial two rounds. Additionally, the actual number of forms remained higher than the figures reported in each of the previous three audits, signifying a positive trajectory in documentation practices.

## Discussion

The aim of this study was to evaluate the effectiveness of the SBCC intervention implemented during the TB REACH wave 9 project to improve documentation of specimen examination request forms by frontline health workers. To the best of our knowledge, this study is the first of its kind in Nigeria that had evaluated SBCC interventions on documentation practices among frontline health workers. Overall, our results demonstrated a consistent and progressive improvement in the accuracy and completeness of recorded information following multiple administrations of the intervention. This underscores the effectiveness of the intervention in improving documentation practices. This result is consistent with results from similar studies outside Nigeria that demonstrated an improvement in documentation among health care workers following a targeted intervention (34–36).

The success of our intervention in enhancing documentation practices can be ascribed to the thoughtful design of the intervention, which systematically tackled several contributing factors identified in the literature associated with subpar documentation. Poor documentation has been identified in some studies as a result of factors such as lack of training, deficient skills, and insufficient awareness and knowledge regarding what and how to document (15, 16, 20). In line with the information and behavioral skills constructs of the IMB model of behavior change, our intervention addressed these issues through multiple training sessions. During these sessions, participants were guided through each variable in the specimen examination form, with explanations highlighting the significance of each variable and outlining the criteria for achieving a complete and accurate record. It is noteworthy that the training sessions were repeated at intervals to reinforce the communicated lessons. This is to address the concern that training on documentation is more often non-existent or at best sporadic (34) and support the recommendation for continuous training on this subject (16).

Our intervention also addressed factors such as poor motivation and attitude of health care workers toward documentation (11). According to the IMB model, motivation for behavior change has both personal and social aspects (33). Enhancing personal motivation requires health workers to understand the advantages of the good documentation and the potential negative consequences if not embraced. This will in turn influence their attitude toward adopting the behavior (18, 22). Through the animated educational video used during the trainings, the impact of poor documentation on the patient and health system was aptly communicated. The link between patient health outcomes, adequate management of scarce resources, effective service delivery by other members of the healthcare team, personal job satisfaction and good documentation was clearly elucidated.

Studies also identified inadequate supervision of healthcare workers, lack of discipline and encouragement from superiors, and shortage of recording materials as demotivators for proper documentation (11, 16, 20). The active involvement of the State and Local Government TB program teams in the intervention, likely provided the social support and motivation health workers needed to embrace proper documentation practices during the period. These teams consistently offered reminders during various meetings and supervisory visits about the importance of thorough documentation. They also followed up on non-adherent health workers and supported to enhance the availability of revised specimen examination forms in healthcare facilities. Their involvement highlighted the priority placed on proper documentation in patient management, emphasizing its importance.

A crucial success factor for our intervention is the systematic and regular audit and feedback implemented throughout the intervention. The absence of a consistent monitoring and evaluation system for documentation practices has been identified as a key contributor to poor-quality documentation (11, 16, 20). The routine audit of specimen examination forms during the intervention played a pivotal role in monitoring the progress of documentation practices, identifying persistent gaps, and determining health workers in need of additional support. This process guided personalized one-on-one follow-up mentoring and highlighted areas for emphasis in subsequent training sessions. The feedback on progress, coupled with subsequent encouragement, also served as motivation for improvement among those performing poorly and reinforcement for those exhibiting commendable practices. This most probably contributed to the progressive improvement observed in follow-up audit rounds for most of the variables reviewed.

Taking into consideration the findings of some studies highlighting overwhelming workload and shortage of staff as contributing factors to poor documentation (15, 18), the result of our intervention demonstrated that these might not be the predominant issues. While increasing staff strength is undeniably important to reducing workload and potentially improving the attention paid to documentation, several challenges with the recruitment of more staff, including inadequate funding and brain drain, especially in low-and middle-income countries may pose a hindrance and contribute to delay in the desired outcome. For immediate results, improving the knowledge and skills of available staff, ensuring their motivation, providing adequate support and instituting effective monitoring and feedback mechanism holds the potential to significantly enhance documentation practices as demonstrated by our study, while concurrently addressing the issue of low staff strength. This assertion is consistent with the findings from a study in Indonesia (11), and a systematic review and meta-analysis estimating the pooled level of documentation practices in Ethiopia (21). These studies highlighted good knowledge, adequate training/education, favorable attitudes, increased confidence and motivation, adequate supervision and availability of documentation guidelines as top factors for quality documentation.

### Limitations of the study

While the study findings offer valuable information on trends and intervention impacts within the selected facilities, they should be interpreted with caution when applied to a broader context. The facilities selected for the audits were not chosen using a probability sampling technique. Consequently, the sample may not have been fully representative of the entire population. The reliance on available specimen examination request forms at the time of audits may have also led to an overrepresentation or underrepresentation of certain documentation practices. Additionally, observer bias may have influenced documentation audits, and healthcare workers might have modified their behavior due to awareness of being monitored (Hawthorne effect).

However, selecting GeneXpert sites, which serve multiple facilities, helped mitigate the limitation of the purposive facility selection by ensuring diverse representation and maximizing impact, as each GeneXpert site processes forms from an average of 20 different health facilities. The use of standardized data collection tool-a structured checklist and objective data source-a specimen examination form, instead of human judgment was intentional to reduce observer bias. Additionally, the study’s longitudinal design further enhances internal validity by tracking changes over time, reducing the risk of temporary response biases influencing the results.

Future studies using randomized controlled designs could validate our findings and assess their generalizability. Such studies should focus on assessing whether the improvements are sustained over time, identifying any emerging challenges, and exploring additional strategies for enhancing sustainability. Qualitative studies involving healthcare workers and policymakers will provide deeper insights into factors that facilitate or hinder lasting change.

## Conclusion

The SBCC intervention markedly enhanced the accurate and comprehensive documentation of specimen examination request forms among frontline healthcare workers. This success was driven by key components, such as repeated training sessions, education emphasizing the benefits of sound documentation practices, consistent supervision, monitoring, feedback, and ensuring a sufficient supply of forms. Together, these elements maximized the effectiveness of the intervention.

These findings provide an evidence base that can inform public healthcare policies and practices. For sustained improvements in documentation and reporting, it is recommended that these enhancements be integrated into standard routine facility protocols. Reinforcement through continuous training and capacity-building initiatives for healthcare workers to promote best practices, and embedding quality assurance mechanisms, such as periodic audits and supervisory feedback, will help maintain high standards and ensure accountability in documentation.

## Data Availability

The raw data supporting the conclusions of this article will be made available by the authors, without undue reservation.
